# Longitudinal study of pregnancy intention and its association with pregnancy occurrence among female sex workers in Benin and Mali

**DOI:** 10.1186/s12978-023-01565-4

**Published:** 2023-01-30

**Authors:** Gentiane Perrault Sullivan, Fernand Aimé Guédou, Fatoumata Korika Tounkara, Luc Béhanzin, Nana Camara, Marlène Aza-Gnandji, Bintou Dembele Keita, Odette Azonnadou, Ismaila Thera, Lisa Avery, Michel Alary

**Affiliations:** 1 Département de Médecine Sociale et Préventive, Université Laval, Québec, Canada; 2 Axe Santé des Populations et Pratiques Optimales en Santé, Centre de recherche du Chu de Québec – Université Laval, Québec, Canada; 3 Dispensaire IST, Centre de Santé Communal de Cotonou 1, Cotonou, Benin; 4 École Nationale de Formation des Techniciens Supérieurs en Santé Publique et en Surveillance Épidémiologique, Université de Parakou, Parakou, Benin; 5 ARCAD∕SIDA, Bamako, Mali; 6 Institute for Global Public Health, Dept. Obstetrics, Gynecology and Reproductive Sciences, Max Rady Medical College of Medicine, University of Manitoba, Winnipeg, Canada; 7 Institut National de Santé Publique, Québec, Canada

**Keywords:** Pregnancy incidence, Pregnancy intention, Reproductive health, Sex workers, Sub-Saharan Africa

## Abstract

**Background:**

The intention of becoming pregnant has an evident impact on the prenatal and postnatal period. For female sex workers (FSWs) in West Africa, among whom pregnancies are frequent as are HIV and sexually transmitted infections, a better understanding of their pregnancy intention and its influence on pregnancy occurrence could help prevent unwanted pregnancies and adverse effects on wanted pregnancies.

**Methods:**

We recruited 330 FSWs in Benin and 322 in Mali and followed them for 12 months. We evaluated their pregnancy intention at recruitment and 6-month follow-up, using a multidimensional prospective measure that we developed. We assessed pregnancy occurrence with a pregnancy test and a retrospective questionnaire at 6 and 12 months. A Cox proportional hazard model was used to estimate the association between intention and pregnancy. We carried out an analysis to take losses to follow-up into account using the inverse of probability of censoring weights and a cluster analysis to corroborate that the multidimensional measure of pregnancy intention fitted the data.

**Results:**

407 FSWs were included in the first 6-month analysis and 284 at 12 months. Mean age was 30.9 years. The pregnancy intention distribution was similar between the two periods: 15.2% in the first period and 16.3% in the second had a positive intention. One out of four were ambivalent and almost 60% (57.7% and 56.3%) had a negative intention. For 38.2% of the FSWs, the intention changed between the two periods. The global incidence rate (to first event) was 19.1 pregnancies per 100 person-years. There was a borderline significant trend (p = 0.0529) of decreased pregnancy incidence with decreasing intention. Compared to positive intention, the adjusted hazard ratio (aHR) for ambivalent and negative intentions were 0.71 [95% confidence interval (95% CI) 0.32–1.60] and 0.46 (95% CI 0.21–1.01), respectively.

**Conclusion:**

The level of pregnancy intention influences its occurrence among FSWs and nearly one out of six wants a baby despite working in the sex trade. Programmatically, early identification of these women could facilitate provision of quality antenatal and postnatal care. Given other health risks associated with sex work this care may decrease potential risks of adverse maternal, fetal and neonatal outcomes.

**Supplementary Information:**

The online version contains supplementary material available at 10.1186/s12978-023-01565-4.

## Plain Language Summary

The intention of becoming pregnant has an evident impact on the prenatal and postnatal period. For female sex workers (FSWs) in West Africa, among whom pregnancies are frequent, a better understanding of their pregnancy intention and its influence on pregnancy occurrence could help prevent unwanted pregnancies and adverse effects of wanted pregnancies. We recruited 330 FSWs in Benin and 322 in Mali and we followed them for 12 months. We evaluated their pregnancy intention at recruitment and at 6-month follow-up. We assessed pregnancy occurrence with a pregnancy urine test and with a retrospective questionnaire asking on pregnancy occurrence at 6 and 12 months. With these data we estimated the association between intention and pregnancy. 407 FSWs were included in the first 6-month analysis and 284 at 12 months. Sixteen percent of the FSWs had a positive intention of having a pregnancy in the next 6 months in both the first 6-month and 12 months. One out of four were ambivalent and almost 60% had a negative intention. For 38.2% of the FSWs the intention changed between the two periods. Women with an ambivalent intention compared to those with positive intention, had 30% less pregnancies whereas for negative intention, women had 54% less pregnancies during the study follow-up. As for any woman, the level of pregnancy intention influences its occurrence. Moreover, nearly one out of six FSWs wants a baby despite working in the sex trade. Programmatically, early identification of these women could facilitate provision of quality antenatal and postnatal care.

**Supplementary Information:**

The online version contains supplementary material available at 10.1186/s12978-023-01565-4.

## Résumé

L’intention de devenir enceinte a un impact évident sur les périodes pré- et post-natales. Les grossesses, tout comme le VIH et les infections transmises sexuellement, sont fréquentes chez les femmes travailleuses du sexe (FTS) en Afrique. Une meilleure compréhension de leur intention de grossesse et l’influence de celle-ci sur leur survenue pourrait aider à prévenir les grossesses non désirées et les effets indésirables lors de grossesses désirées. Des FTS ont été suivies pendant 12 mois (N = 330 Bénin - N = 322 Mali). L’intention d’avoir une grossesse a été évaluée au recrutement et à 6 mois, à l’aide d’une mesure multidimensionnelle prospective développée pour l’étude. La survenue de grossesse a été mesurée à l’aide d’un test d’urine et d’une question rétrospective à 6 et 12 mois. Un modèle des risques proportionnels de Cox a été utilisé pour estimer l’association entre l’intention et la grossesse. Un poids égal à l’inverse de probabilité de sélection a été appliqué pour prendre en compte les femmes perdues au suivi et une analyse hiérarchique par grappe pour valider la mesure de l’intention a été effectuée. 407 FTS (âge moyen 30.9 ans) ont été incluses dans la première période d’analyse et 284 dans la deuxième. Au cours de ces deux périodes, un peu plus de 16% des FTS avaient une intention positive. Une FTS sur 4 était ambivalente et près de 60% avaient une intention négative. Pour 38.2% des FTS, l’intention a changé entre les deux périodes. Le taux d’incidence était de 19.1 grossesses par 100-personne-années. Comparées aux FTS avec une intention positive de grossesse, celles ambivalentes ou avec une intention négative, ont eu moins de grossesses, respectivement 30% [rapport de risque ajusté (RRa) = 0.71, intervalle de confiance à 95% (IC 95%) 0.32–1.60) et 54% (RRa = 0.46, IC 95% 0.21–1.01) moins de grossesses. Chez les FTS l’intention d’avoir ou non une grossesse en influence l’occurrence. De plus, près d’une femme sur six désire une enfant dans les six prochains mois malgré la pratique du travail du sexe. Identifier ces femmes et les accompagner lors de la conception et des périodes pré- et post-natales pourrait aider à réduire les effets indésirables.

## Background

In West Africa, between 1 and 4% of adult women are involved in transactional sex [1]. Within this marginalized population, unmet contraceptive needs are frequent [2] as are pregnancies [3–6]. FSWs face a high burden of unplanned pregnancies [7–9] that increase their stigmatization and the financial burden they face, while negatively impacting their work [10]. It is a real priority for female sex workers (FSW) to avoid unintended pregnancies [11] but it is also important for a portion of them to become mothers [12], even while practicing sex work [13, 14]. The intention surrounding pregnancy is of utmost importance. Indeed, because the intention of becoming pregnant may have a strong impact on the prenatal period [15], the use of antenatal care services [16] and the risk of stillbirths and postnatal depression, especially in low income countries [17], the intention surrounding pregnancy can potentially prevent or exacerbate adverse outcomes that could occur in the pre- and postnatal periods.

Unintended pregnancies are associated with a higher level of maternal mortality and morbidity [18] and have multiple adverse health consequences [19, 20]. In West Africa, where therapeutic abortion is mostly illegal, unsafe abortions are responsible for 10% of all maternal deaths [21]. However, only a portion of unintended pregnancies lead to abortion.

For FSWs deciding to bring their pregnancy to term, humiliation, guilt and stigma surrounding pregnancies within sex work practice make it difficult for them to access sexual and reproductive health care services [22] and prenatal care [23]. FSWs are at high risk of sexually transmitted infections (STIs) [24], including HIV [25, 26], and the process of becoming pregnant puts them at higher risk of acquiring such infections and enhances the risk of mother-to-child transmission [27]. Therefore, FSWs report having adverse pregnancy outcomes: stillbirths, and serious health problems, such as neonatal death, low birthweight, and prematurity [28, 29] which could be avoided with access to sexual and reproductive health care services. Regardless of these risks and challenges, becoming pregnant can also be a means for FSWs to be respected as a mother, recognized by other women [2, 30], and secure a relationship with a partner [31]. Despite all these considerations, the incidence of unintended pregnancies among FSWs is yet to be estimated, given the scarce number of studies assessing pregnancies and an even lower number assessing the intention surrounding those pregnancies [8]. The limited research investigating pregnancy intention mostly targeted FSWs living with HIV [32–34] and minimal attention has been given to safe pregnancy planning [35].

To assess the incidence of unintended and wanted pregnancies in FSWs, pregnancy intention needs to be considered. Pregnancy intention is a complex and difficult concept to estimate [36]. A unidimensional measure of pregnancy intention does not represent well the complexity of the factors involved in the women’s intention [37]. The intendedness of pregnancy is influenced by the context in which the woman evolves [38]. The use of a multidimensional conceptual model [39, 40] prospectively assessed provides a more nuanced appraisal of pregnancy intention [41]. For instance, in Bangladesh, the further away the idea of having a baby was, the more prevalent the positive intention of having a baby was reported (soon or now 2.9%, less than 12 months 3.8%, less than 24 months 14.8% and after marriage 27.1% [13]). Similarly, FSWs living with HIV in Kenya were asked about fertility desire (Do you want any/more children?) and fertility intention (Are you trying to get pregnant now?) and a 15% discrepancy was observed (25.5% a positive fertility desire vs 10.2% had a positive fertility intention) [33]. In light of these observations, the use of a prospective multidimensional tool that considers the changing context in which FSWs evolve appears to be needed to validly assess pregnancy intention in this population.

The main objective of this research was to determine if FSWs want to have children during sex work practice. We reached this goal through four specific objectives. We first developed a construct that measures the concept of pregnancy intention and verified this construct with a cluster analysis. Secondly, we described the variation in pregnancy intention between two follow-up periods at 6-month intervals. Thirdly, we characterized the occurrence of pregnancy for two different definitions of pregnancy intention as well as the occurrence of intended and unintended pregnancies. Fourthly, we investigated the association between intention and having at least one pregnancy during the overall follow-up period.

## Methods

The present study was combined with another one investigating cervical cancer and human papillomavirus (HPV) infection among FSWs [42]. Peer educators (PEs) selected from non-governmental organizations (NGOs) working with FSW in the field of HIV/STI prevention conducted outreach activities at sex work venues (bars, brothels, hotels, etc.) to raise awareness on cervical cancer screening. The PEs in Cotonou, Benin, and Bamako, Mali, were well known to FSWs. Following the outreach activities, women practicing sex work who wished to participate in the study were referred by PEs to FSW-friendly clinics *Dispensaire IST* (DIST) in, Benin, and *Clinique des Halles* run by ARCAD/SIDA, a national Non-Governmental Organization (NGO), in Mali, for cervical cancer screening. After participating in the HPV study, they were offered to participate in the study on pregnancy intention (see details in Perrault Sullivan et al. [43]). Three hundred and thirty women were recruited in Cotonou, Benin, between March and June 2017 and 322 in Bamako, Mali, between November 2017 and February 2018 for the study on reproductive health. Women were evaluated at recruitment and had two follow-up visits after six and twelve months.

### Inclusion criteria

For both sites (Cotonou, Benin and Bamako, Mali) FSWs had to be at least 18 years old and to provide written informed consent to participate in the study on reproductive health. They also needed to have been involved in sex trade for at least 6 months.

### Data collection

All the data regarding sociodemographic characteristics as well as the HIV and pregnancy test results were extracted from the HPV study dataset. Sociodemographic characteristics were collected only at recruitment. In addition, a quantitative questionnaire was administered during a face-to-face interview asking questions on reproductive history, pregnancy intention and contraception at recruitment and each follow-up visit, at 6 and 12 months. Finally, a pelvic examination and a urine pregnancy test were carried out by a physician at each study visit.

### The concept of pregnancy intention

We wanted to use a prospective measure of pregnancy intention since retrospective measures may underestimate the frequency of unintended pregnancy [44]. To our knowledge, no validated questionnaire exists to prospectively evaluate pregnancy intention in the target population. The *London Measure for Unplanned Pregnancy* (LMUP) is a validated questionnaire that retrospectively evaluates the planning surrounding a pregnancy [45]. We used this validated tool to help build our questionnaire. We wanted to respect the three aspects that define intention in the LMUP: the *context*, the *stance*, and the *behavior* and the six components included in those aspects (two components in each aspect) [46].

The two components included in the first aspect (the *context*) are the personnel circumstances/timing and the partner influences. The second aspect, the *stance*, comprises the desire of pregnancy and expressed intention and finally, the third aspect, the *behavior*, includes the pre-conceptual preparation and the contraceptive use.

The intention entails a desire for an outcome and a belief that a certain action will bring it about [47]. To measure the intention, we created six questions (the exact wording for each question is available in Additional file 1) inspired by the LMUP questionnaire that cover five of the six components included in the three aspects that define intention (contraceptive use was not included in our questions. The arguments supporting this decision are below). Multi-item measures compared to single-item measures allows to acquire a more nuanced assessment of pregnancy intention [41, 48]. The answers to these six questions were used to create the intention categories: positive, ambivalent, and negative intentions.

For the first aspect, the *context*, we measured the personal circumstances. To do so, we used the gap between the ideal family size and the number of biological children that a woman already had. In addition, we used the perceived timing for a pregnancy (good timing, ok but not a good timing, bad timing).

For the second aspect, *the stance*, we measured pregnancy desire with the following question: ‘’Do you want to be pregnant in the next 6 months?’’ and the expressed intention with: ‘’How important is it for you to avoid a pregnancy in the next 6 months?’’.

Finally, for the third aspect, *the behavior* aspect, we included the pre-conceptual preparation. We asked the FSWs whom they planned to conceive with (client, husband, boyfriend or other) and if they had discussed that possibility with him (no discussion, discussion and disagreement or discussion and agreement) which also covers the partner influence of the second aspect (*context*).

We did not include contraception use in this third (*behavior*) aspect of the intention for two reasons. First, usage of modern contraception is really low among FSWs in the two countries where we recruited participants, especially in Benin [6], and condoms are generally not used with non-paying partners/boyfriends. This lack of condom use is linked predominantly to preference to distinguish between personal and professional life [31, 49–51], and not generally with desire for pregnancy planning. Secondly, in an African population, the LMUP questionnaire’s aspect of planning that relates to contraception use did not correlate well with the evaluation of pregnancy planning [52]. In addition, the LMUP questionnaire needed to be adapted to the context of sex work [53]. Given those aspects, we decided to inquire on the planning of the pregnancy instead. We decided to inquire about the discussion of a possible pregnancy with the potential father. Finally, instead of including contraception use in the measure of intention we included the use of hormonal contraception in our analysis of the effect of intention on pregnancy occurrence (see Additional file 1).

To validate our choices, we used a cluster analysis. We wanted to ascertain that the questions we regrouped to evaluate the three aspects of the intention were measuring the same aspect of the intention and that they were mutually exclusive [54].

### Independent variables

As previously mentioned, we defined three levels of pregnancy intention based on six different questions that defined three aspects of the pregnancy intention (see Additional file 1). These levels were positive, ambivalent, and negative intention. Because we wanted to compare different measures of pregnancy intention for our third objective, we also used a dichotomized variable for the intention (positive intention vs. negative intention). This independent variable was based on the question “do you have the intention of becoming pregnant in the next 6 months?” A “yes” answer refers to a positive intention and a “no” answer, to a negative intention. Questions regarding intention were asked prospectively at recruitment and at the six-month follow-up visit.

### Dependent variable

We defined the dependent variable as having at least one pregnancy during the study period. To evaluate the number of women meeting this definition, we combined two different measures. First, we used a retrospective question asked at six and twelve months: have you been pregnant since your last study’s interview? Secondly, we used the results of the pregnancy test administered at six and twelve months. Women who were pregnant at recruitment were excluded and women who declared a pregnancy or had a positive pregnancy test at 6 months were not included in the following period. Finally, many questions were asked in the questionnaire to make sure we count every pregnancy only once and that we attribute the time at risk properly. We asked women about the total number of pregnancies they had, the issue of each of the pregnancy and from whom they were. We asked these three questions for the period preceding sex work practice and the period during sex work practice. We also asked the same three questions for their entire lifetime. By comparing all the answers and making sure the number were compatible with each other were the same we were able to verify all the answers.

### Covariates and sociodemographic characteristics

The sociodemographic characteristics were collected at recruitment and 6-month follow-up. Questions regarding personal characteristics (ex. age, country of origin, religion, education, marital status, having a boyfriend, number of biological children, etc.), sex work practice (ex. age at sex work debut, duration of involvement in sex work practice, money received for last paying sex, etc.) and sexual health (HIV status, pregnancy, condom use, use of hormonal contraception, etc.) were asked as part of the HPV research, and were available for our analyses.

### Missing data

The women who had no information on intention were removed from the period where they did not provide this information. They could have been included in the following period if at that time, the information on the intention was available. We also could not include women who were lost to follow up. We initially compared the characteristics of women who came back with those lost to follow up. Then, given that there were some differences between these two groups, we used inverse probability of censoring weights (IPCW) to correct for biases that may be induced by losses to follow-up [55].

### Statistical analyses

We used descriptive analyses (frequencies, means values and standard deviations) to present the characteristics of the participating FSWs. We also show a comparison (using Pearson chi-square) between the women lost to follow-up and those retained in the study for the two periods. These comparisons provide information on possible selection biases introduced by losses to follow-up.

The cluster analysis was an iterative process. We started with a model including the six variables inspired by the LMUF and identified a priori. The procedure begins with a single cluster then divides the existing one into two sub-clusters until it reaches the maximum number of clusters. That process produces a hierarchy of disjoint clusters [56]. We tried the procedure with no specified restriction for the number of clusters and with three pre-defined clusters, as supported by the decision made a priori. To decide which cluster fits best the data, we used two markers. The first marker is the 1-R^2^ ratio. It represents the ratio between the proportion of the cluster’s variance explained by the variable divided by the proportion of the nearest cluster variance explained by the same variable. The variable with the smallest 1-R^2^ ratio was subsequently removed from the cluster analysis. In addition, the proportion of the variance explained by the identified clusters was used to choose the best variable grouping that explained the intention [57–59]. We selected the best clusters based on these two indicators.

A logistic regression model was fitted to the data to estimate for each observation, the probability of being lost to follow-up in each respective period. This procedure was carried out independently for the two periods. The covariates included in the logistic regression model were selected a priori. They were the ones who could predict losses to follow-up and the outcome of interest (having at least one pregnancy during the last 6 months) [55]. The covariates that were included in the logistic models were pregnancy intention, age, being a migrant, religion, education, matrimonial status, the number of biological children, the use of condom with a boyfriend and the HIV status [60–62]. At the end of this process, weights based on the inverse probability of censoring (IPCWs) were attributed to each woman that was not lost to follow-up to ensure that the final sample represented the one we had recruited, including those lost to follow-up.

We computed incidence rates of pregnancy (to first event) overall and according to the two intention classifications. Exposure time was determined as the entire period for women who learned they were pregnant with the pregnancy test at the follow up-visit or when censored for those who never got pregnant during follow-up. For women who declared a pregnancy between two follow-up visits, half of the period was considered at risk. A Cox proportional hazard model was used to assess the association between intention and pregnancy occurrence, with intention as a time-varying exposure variable. This analysis was adjusted for baseline factors associated with pregnancy (identified previously) [6, 8] and known as potentially associated with intention (age, migrant status, religion, number of years as a sex worker, number of biological children, marital status, condom use with boyfriend, hormonal contraception and HIV status). Manual backward selection was carried out with a ten percent threshold to get to the final model [63]. Finally, the hazard ratios (HRs) were computed with and without the IPCWs that we calculated with the logistic regressions. We performed all the analyses using SAS 9.4 (SAS Institute Inc. Cary, NC).

### Ethical considerations

Each participant provided a signed informed consent, and no nominal information was reported on the questionnaire. The participants received a monetary compensation to cover their transport fees and the possible loss of income due to their participation (approximately 5 US dollar in Mali and 2 US dollar in Benin). The study was approved by the ethics committees of the CHU de Québec – Université Laval (Québec, Canada) and of the School of Medicine of Bamako, Mali, as well as by the National Health Research Ethics Committee in Benin.

## Results

### Missing data and losses to follow-up

**Fig. 1:**
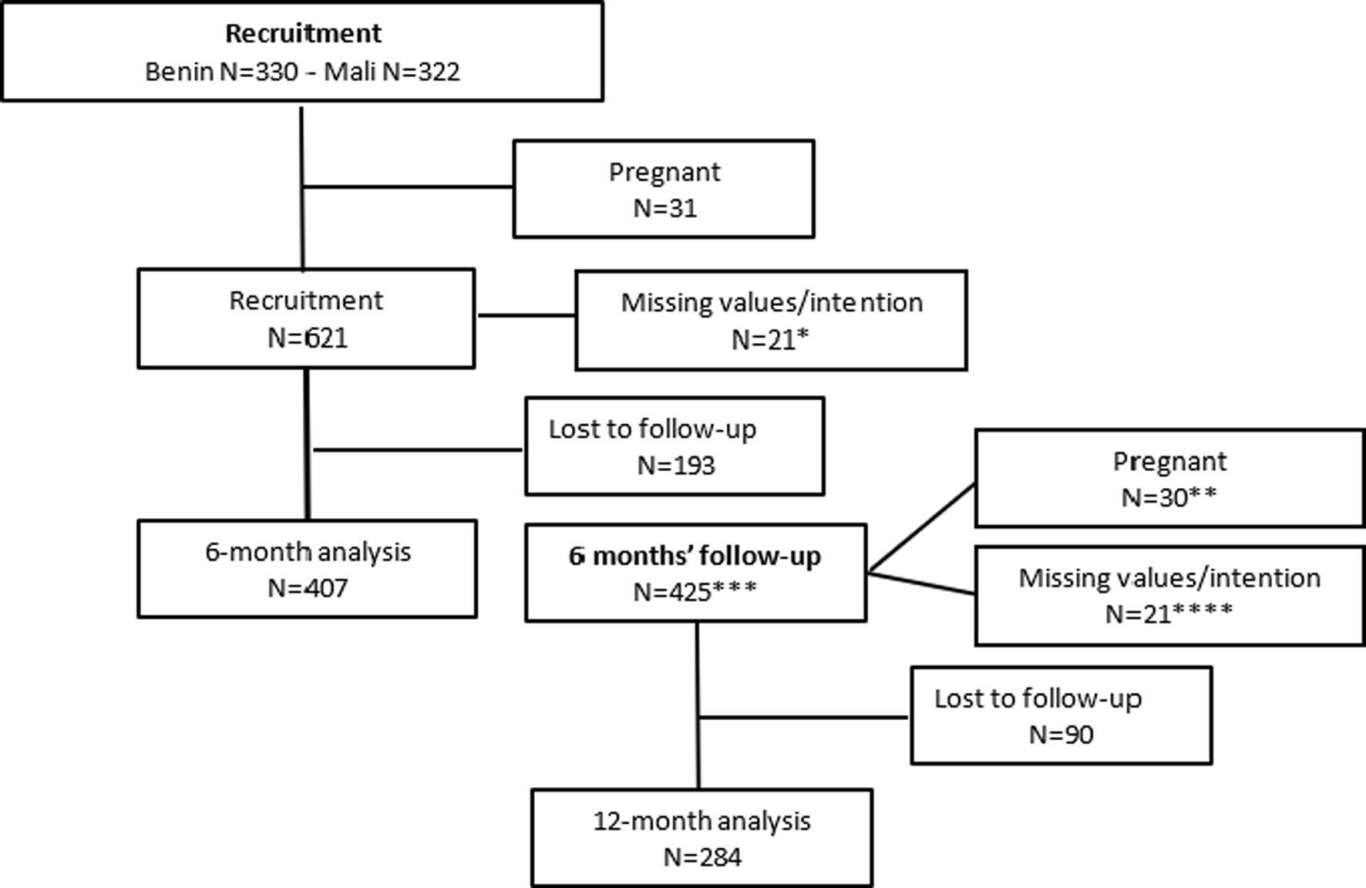
Flow chart of recruitment and follow-up. * 3 women with missing value for the intention were also lost to follow-up and are not included in the lost to follow-up box. ** Had at least one pregnancy during the first 6 months. *** Including the 407 women included in the 6-month analysis and 18 women with missing values at recruitment but still retained at the 6-month follow-up visit. **** 13 women with a missing value for the intention were also lost to follow-up and are not included in the lost to follow-up box

A total of 652 FSWs were recruited in the two countries (n = 322 in Mali and n = 330 in Benin), of whom 31 (n = 18 in Mali and n = 13 in Benin) were pregnant and excluded from these analyses because it was not possible to measure their prospective intention regarding a pregnancy. Given the missing values for pregnancy intention at recruitment and at 6 months, and the losses to follow-up in the first and second 6-month follow-up period (Fig. 1), we could thus include a total of 407 (n = 190 in Mali and n = 217 in Benin) and 284 (n = 128 in Mali and n = 156 in Benin) women for the first and second period analyses, respectively. For the incidence rates and the Cox proportional hazard model, all women with at least one follow-up visit with a prior measure of intention were included (n = 416). In fact, among the 425 FSWs who had at least one follow-up visit, five had a missing value for the intention and were lost to follow up and four had a missing value for the intention at both the six- and twelve-month follow-up.

### Cluster analysis

We tried eight different models before reaching the best fit. The first model included the six variables identified a priori*.* The final model chosen (option 8 in Additional file 2) was the one for which the highest percentage of the variance in the data was accounted for (95.83%) and for which the separation between each variable and the nearest clusters was optimal. It included four variables distributed in three clusters (Table 1).

**Table 1:**
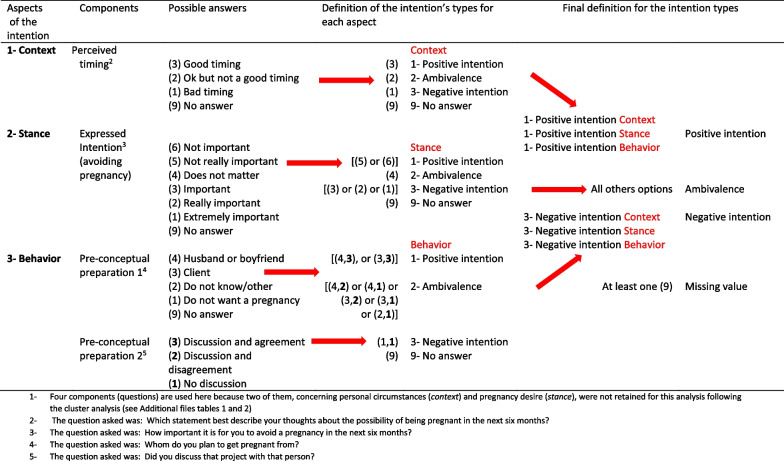
Final definition of the three types of pregnancy intention based on the answers to the four questions identified through the cluster analysis^1^

To be classified as having a positive intention, FSWs had to answer in favor of a future pregnancy in the three clusters for all the questions. To be included in the negative intention’s category the four questions needed to be in support of avoiding a pregnancy. Finally, women who had discrepancies in their answers were classified as ambivalent. For example, if a FSW answered that it was a good timing to be pregnant, it was not important to avoid a pregnancy but she planned to get pregnant from a man that she had not met previously*,* she was considered as ambivalent (Table 1).

### Descriptive analyses

We had the information on the baseline characteristics for 600 FSWs without missing values on intention at baseline (Table 2). Mean age was 30.9 [standard deviation (SD): 10.2] and 46% were migrant. Women who were lost to follow-up at each period were mostly between 25 and 29 years old. We also lost more migrant women during the second period (p-value = 0.0027) while we observed no difference during the first period (p-value = 0.3310). More than 70% of the FSWs had no secondary school education and 42.2% were Muslim. Less than ten percent disclosed being married or in a non-marital relationship and 63% had a boyfriend. The women lost to follow-up during the first period were more often single (p-value = 0.0015) and during the second period it was women with a boyfriend that were not returning to the study visit (p-value = 0.0380). Almost 70% of FSWs had at least one biological child for a mean of 1.5 children per women. Women with no children were more likely to drop out of the study during the first 6 months (p-value = 0.0158).

**Table 2:** Sociodemographic and sex work characteristics of female sex workers with available data on pregnancy intention for each study period

Sociodemographic variables		First period				Second period			
		Recruitment (n = 600)	Lost to follow-up (n = 193)	6-month analysis (n = 407)		6-month follow-up (n = 374)	Lost to follow-up (n = 90)	12-month follow-up (n = 284)	
	Mean (SD)	Frequency (%)			p-value*	Frequency (%)			p-value*
Age	30.9 (10.2)								
< 25		211 (35.2)	73 (37.8)	138 (33.9)		115 (30.8)	33 (36.7)	82 28.9)	
25–29		132 (22.0)	59 (30.6)	73 (17.9)		62 (16.6)	21 (23.3)	41 (14.4)	
30–34		83 (13.8)	27 (14.0)	56 (13.8)		53 (14.2)	10 (11.1)	43 (15.1)	
≤ 35		174 (29.0)	34 (17.6)	140 (34.4)	**< 0.0001**	144 (38.5)	26 (28.9)	118 (41.5)	**0.0413**
Migrant									
No		325 (54.2)	99 (51.3)	226 (55.5)		209 (55.8)	38 (42.2)	171 (60.2)	
Yes		275 (45.8)	94 (48.7)	181 (44.5)	0.3310	165 (44.1)	52 (57.8)	113 (39.8)	**0.0027**
Religion									
Muslim		252 (42.2)	84 (44.2)	168 (41.3)		146 (39.0)	33 (36.7)	113 (39.8)	
Catholic		134 (22.5)	38 (20.0)	96 (23.6)		95 (25.4)	23 (25.6)	72 (25.4)	
Other		211 (35.3)	68 (35.8)	143 (35.1)	0.5998	133 (35.6)	34 (37.8)	99 (34.9)	0.8456
Missing		3	3						
Education									
Unschooled or Koranic school		200 (33.5)	64 (33.7)	136 (33.4)		132 (35.4)	26 (28.9)	106 (37.5)	
Primary		238 (39.9)	73 (38.4)	165 (40.5)		149 (40.0)	42 (46.7)	107 (37.8)	
Secondary		121 (20.3)	40 (21.1)	81 (19.9)		71 (20.3)	15 (16.7)	56 (19.8)	
Superior		38 (6.4)	13 (6.8)	25 (6.1)	0.9537	21 (6.4)	7 (7.8)	14 (5.0)	0.2531
Missing		3	3			1		1	
Marital status									
Married or non-marital relationship		51 (8.5)	12 (6.3)	39 (9.6)		33 (8.8)	7 (7.8)	26 (9.2)	
Divorced, separated or widowed		214 (35.9)	52 (27.4)	162 (39.8)		162 (43.8)	35 (38.9)	127 (44.7)	
Single		332 (55.6)	126 (66.3)	206 (50.6)	**0.0015**	179 (47.9)	48 (53.3)	131 (46.1)	0.4905
Missing		3	3						
Has a boyfriend									
Yes		369 (62.9)	116 (63.0)	253 (62.8)		228 (61.4)	63 (70.8)	166 (58.5)	
No		218 (37.1)	68 (37.0)	15 (37.2)	0.9510	143 (38.5)	26 (29.2)	117 (41.5)	**0.0380**
Missing		13	9	4		3	1	1	
Number of biological children	1.5 (1.5)								
None		198 (33.0)	77 (39.9)	121 (29.7)		107 (28.6)	30 (33.3)	77 (27.1)	
1		167 (27.8)	55 (28.5)	112 (27.5)		100 (26.7)	25 (27.8)	75 (26.4)	
≥ 2		235 (39.2)	61 (31.6)	174 (42.8)	**0.0158**	167 (44.7)	35 (38.9)	132 (46.5)	0.3957
HIV status									
Positive		131 (21.8)	37 (19.2)	94 (23.1)		91 (24.5)	13 (14.6)	78 (27.6)	
Negative		467 (77.8)	156 (80.8)	311 (76.8)	0.2642	281 (75.5)	76 (85.4)	205 (72.4)	**0.0131**
Missing		2		2		2	1	1	
At least one pregnancy									
Yes				39 (6.5)				24 (7.2)	
No				368 (61.3)				221 (66.0)	
Total number of pregnancies (mean)									
				43 (1.1)				25 (1.0)	
Pregnancy intention									
Positive		97 (16.2)	35 (18.1)	62 (15.2)		61 (16.4)	15 (16.7)	46 (16.3)	
Ambivalent		158 (26.3)	48 (27.0)	110 (27.0)		100 (26.3)	22 (24.4)	78 (27.5)	
Negative		345 (57.5)	110 (57.0)	235 (57.7)	0.6295	213 (57.3)	53 (58.9)	160 (56.3)	0.8519
Pregnancy intention (dichotomic)									
Positive		166 (27.7)	62 (32.1)	104 (25.6)		94 (25.1)	21 (23.3)	73 (25.7)	
Negative		434 (72.3)	131 (67.9)	303 (74.5)	0.0928	280 (74.9)	69 (76.7)	211 (74.3)	0.6514
Age at sex work debut (years)									
≤ 17		124 (21.1)	38 (20.5)	86 (21.43)		73 (19.7)	21 (23.6)	52 (18.4)	
18–21		154 (26.2)	60 (32.4)	94 (23.3)		81 (21.8)	19 (21.4)	62 (22.0)	
22–25		107 (18.2)	39 (21.1)	68 (16.8)		59 (15.9)	14 (15.7)	45 (16.0)	
26–29		63 (10.7)	17 (9.2)	46 (11.4)		44 (11.9)	14 (15.7)	30 (10.6)	
≥ 30		141 (23.9)	31 (16.8)	110 (27.2)	**0.0193**	114 (30.7)	21 (23.6)	93 (33.0)	0.3612
Missing		11	8	7		3	1	1	
Duration of involvement in sex work (years)									
≤ 1		109 (18.3)	42 (22.2)	67 (16.5)		64 (17.0)	19 (21.1)	45 (15.8)	
2		141 (23.7)	55 (29.1)	86 (21.1)		68 (18.0)	21 (23.3)	45 (15.9)	
3–4		146 (24.5)	40 (21.2)	106 (26.0)		97 (25.7)	22 (24.4)	75 (26.4)	
5–9		131 (22.0)	36 (19.1)	95 (23.3)		92 (24.4)	18 (20.0)	74 (26.1)	
≥ 10		69 (11.6)	16 (8.5)	53 (13.0)	**0.0334**	56 (14.9)	10 (11.1)	45 (15.9)	0.2449
Missing		4	4	0					
Money received for last payed sex (FCFA)^©^	3616.6 (4082.0)								
≤ 1500		133 (22.3)	31 (16.3)	102 (25.1)		106 (28.3)	24 (26.7)	82 (28.9)	
1501—2000		139 (23.4)	48 (25.3)	91 (22.4)		83 (22.2)	18 (20.0)	65 (22.9)	
2001—5000		272 (45.6)	94 (49.5)	178 (43.7)		156 (41.7)	42 (46.7)	114 (40.1)	
> 5000		53 (8.9)	17 (9.0)	36 (8.9)	0.1189	29 (7.8)	6 (6.7)	23 (8.0)	0.7404
Missing		3	3						
Condom use with boyfriend^©©^									
No boyfriend		162 (27.6)	56 (30.4)	106 (26.3)		95 (25.6)	28 (31.5)	67 (23.8)	
Never/Not always		176 (30.0)	50 (31.3)	126 (31.3)		113 (30.5)	28 (31.5)	85 (30.1)	
Always		31 (5.3)	10 (5.4)	21 (5.2)		20 (5.4)	7 (7.9)	13 (4.6)	
No sexual relation		218 (37.1)	68 (37.0)	150 (37.2)	0.6820	143 (38.5)	26 (29.2)	117 (41.5)	0.1339
Missing		13	11			3	1	1	
Use of hormonal contraception^©©©^									
Yes		146 (24.4)	46 (23.8)	100 (24.6)		71 (19.0)	22 (24.4)	49 (17.3)	
No		453 (75.6)	147 (76.2)	306 (75.4)	0.8320	303 (81.0)	68 (75.6)	235 (82.8)	0.1296
Missing		1	1						

*According to Pearson chi-square comparing women lost to follow-up with those included in the analysis

SD: Standard deviation

^©^FCFA (1 US dollars $\sim 500 FCFA$)

^©©^During the last 7 days

^©©©^For the next six months including pills, intrauterine device, injectable and implants

A little more than 20% of the women were HIV positive (21.8% at baseline and 24.5% at month 6). It was during the second half of the study that more HIV-negative women did not come back for the follow-up visit (p-value = 0.0131). Women reported starting selling sex at a mean age of 25 years old (SD 8.9) and had been involved in the sex trade for 4.6 years on average (SD 4.30). Women who were lost to follow up during the first period were younger (p-value = 0.0193) and had been involved in the sex trade for a shorter period (p-value = 0.0334) than those who came back at 6 months. There was no discrepancy between the two follow-up periods regarding the amount of money received for the last payed sex (~ 7$ US dollars) and contraception use. Only 5% of the FSWs always used a condom with their boyfriend during the last 7 days and less than one out of four planned using hormonal contraception during the next 6 months.

### Pregnancy intention and its variation

The distribution for the three-category pregnancy intention was similar between the two periods. FSWs who had a positive intention for the next 6 months represented around 15% (15.2% at baseline and 16.3% at month 6) of the women. Women who clearly did not plan to become pregnant accounted for almost 60% (57.7% at baseline and 56.3% at month 6) and nearly 30% were ambivalent (27.0% at baseline and 27.5% at month 6). When evaluating the intention with the usual categories (positive and negative intention), one out of four women (25.6% at baseline and 25.7% at month 6) wanted to have a baby in the next six months, without significant differences between women lost to follow-up and the others (p-value = 0.0928 and p-value = 0.6514) (Table 2).

While the proportion for each type of intention between the two periods were similar, the intention for each woman was not stable over time. One out of three women, for whom the intention was measured for the two periods (123/362), changed their perspective regarding their intention. 8.3% of the FSWs switched completely their intention, changing from a positive intention to a negative or vice versa. For two thirds of the women, the intention was stable over the twelve-month period. The most stable type of intention was the negative intention at 44% (160/362). A positive intention was stable in 8% and ambivalence in 14% (Table 3).

**Table 3:** Female sex workers’ variation of intention between the two periods for the 3-category intention

Period 1—First six months–	Period 2—Second six months–	Recruitment (n = 621) Frequency (%)	
Stable intention			239 (66.0)
Positive intention	"	30 (8.3)	
Ambivalence	"	49 (13.5)	
Negative intention	"	160 (44.2)	
Variable intention			123 (34.0)
Positive intention	Ambivalence	14 (3.9)	
Positive intention	Negative intention	10 (2.8)	
Ambivalence	Positive intention	9 (2.5)	
Ambivalence	Negative intention	35 (9.7)	
Negative intention	Positive intention	21 (5.8)	
Negative intention	Ambivalence	34 (9.4)	
At least one missing value*			259**
Positive intention	–	43	
Ambivalence	–	65	
Negative intention	–	130	
–	Positive intention	1	
–	Ambivalence	3	
–	Negative intention	8	
–	–	9	

*For either the first period or the second period or both

**Includes all women who were lost to follow-up (n = 196)

### Pregnancy occurrence and cumulative pregnancy incidence for the two intention classifications

During the entire study, 63 women had at least one pregnancy, 39 during the first period and 24 during the second one. Three women had more than one pregnancy during the first 6 months (Table 2). Two of them had two pregnancies and one woman declared three abortions, for a total of 43 pregnancies. One woman declared two pregnancies during the second period for a total of 25 (Table 2). No woman reported a pregnancy during both the first and the second period. Over the two follow-up periods, 47 FSWs had a positive pregnancy test. Of those women, 42 (89.4%) also reported that they were pregnant. In addition, 21 pregnancies occurred in the preceding months and were self-declared.

With regards to the two-category intention, 13% (14/104) of the FSWs with a positive intention had a pregnancy during the first period and 15% (11/73) during the second. Eight (25/303) and six percent (13/210) of the women with a negative intention experienced a pregnancy in the first and second periods, respectively. For the three-category intention, about seven percent of the FSWs with a negative intention had a pregnancy during each period (7.2% (17/235) and 7.5% (12/159)). For the ambivalent intention, the cumulative incidence substantially decreased between the two periods (12.7% (14/110) vs 3.8% (3/78)), whereas it increased for the positive intention (12.9% (8/62) vs 19.6% (9/46)). Among the four women with more than one pregnancy, none had a positive intention, three were ambivalent (including the one reporting three abortions) and two had a negative intention.

### Incidence rate and association between intention and having at least one pregnancy

The global incidence rate of having at least one pregnancy during the entire study period was 19.1 per 100 person-years. For the two-category intention, the incidence rates were 30.0 and 15.7 pregnancies per 100 person-years for FSWs with a positive intention and those with a negative intention, respectively. For the three-category intention, the incidence per 100 person-years was 15.2 for women with a negative intention. Women with an ambivalent intention had a higher incidence with 18.9 pregnancies per 100 person-years. Finally, FSWs with a positive intention had 34.2 pregnancies per 100 person-years.

In the Cox proportional hazard model, when we compared the incidence rate of pregnancy between women who had an ambivalent intention and those with positive intention, we observed that they had 30% less pregnancies (aHR 0.71, 95% CI 0.32–1.60). The difference between women with a positive intention and those who do not want a pregnancy is larger, the latter having 54% less pregnancies (aHR 0.46, 95% CI 0.21–1.01). We observed, with an almost statistically significant test for linear trend (p-tend = 0.0529), that the incidence rate of pregnancy was in accordance with the intention. The more desirable the pregnancy is, the greater the incidence rate. For the two-category intention, women with a negative intention had two times less pregnancies than those with a positive intention (aHR 0.53, 95% CI 0.28–1.01) (Table 4).

**Table 4:** Incidence rate of pregnancy (to first occurrence) according to intention level among 416 female sex workers over twelve months of follow-up

	Pregnancies/Per years at risk	Incidence per 100 person-years°	Crude		Adjusted*		Adjusted/IPCW**		P-trend***
			HR°°	95% CI	HR	95% CI	HR	95% CI	
	63/330.5	19.06							
3-category intention									
Positive intention	17/49.8	34.17	1		1		1		
Ambivalence	17/90.0	18.89	0.55	0.28–1.09	0.67	0.30–1.47	0.71	0.32–1.60	
Negative intention	29/190.8	15.20	0.44	0.24–0.82	0.46	0.21–0.99	0.46	0.21–1.01	0.0529
2-category intention									
Positive intention	25/82.3	30.40	1		1		1		
Negative intention	38/248.3	15.31	0.50	0.30–0.84	0.51	0.27–0.98	0.53	0.28–1.01	-

*Adjusted for country, age, migrant status, religion, number of biological children, duration of involvement in sex work, condom use with a boyfriend and hormonal contraception

**Model adjusted with IPCW, Inverse probability of censoring weight

***p-value, test for linear trend for the model adjusted with IPCW

°Time to first pregnancy analysis

°°Hazard ratio of having at least one pregnancy according to intention level

## Discussion

FSWs are a marginalized population but have the same rights and desires as other women. To our knowledge, no previous study on pregnancy incidence stratified by intention had ever been conducted prior to this one, despite the importance of this issue was lacking for this population [64] and to our knowledge has never been done in this population. We evaluated whether FSWs wanted to have children while practicing sex work and what was the distribution of pregnancy occurrence according to intention levels. When we questioned the FSWs about their pregnancy intention, 16% of them wanted a pregnancy in the next 6 months. The incidence of pregnancy was high during the study period with 19 pregnancies per 100 person-year and, during each 6-month period studied, 7% of women who did not want to become pregnant did. For almost 40% of them the intention changed between the two periods, and we observed a borderline significant decreasing trend in pregnancy incidence with decreasing intention level.

Approximately 16% of the women clearly wanted a pregnancy in the next 6 months and that prevalence was the same for the two 6-month periods. Other studies have found the same level of positive intention when intention was measured on a short period of time [32, 33]. Motherhood is associated with lesser desire to conceive [65] and the vast majority of pregnancies occurred before sex work practice [43]. In our study population, 70% of the FSWs had at least one biological child. This is similar to figures reported for women who sell sex elsewhere in sub-Saharan Africa [26, 28]. We did adjust for the number of biological children and the incidence rate of pregnancy in women with a positive intention was 34 pregnancies per 100 person-years, twice higher than among women with a negative intention. FSWs have the same desire when it comes to future fertility as other women. To accompany FSWs into fulfilling their fertility desires, we need to consider that sex work practice is associated with a lot of risks [66] and many of those women engage in sex work practice during pregnancy [67] and wish to have children [14]. These women face multiple challenges working while pregnant: violence from clients, difficulties findings clients, fear of HIV acquisition and transmission [68]. Even though mothers may have more access to health care services by seeking antenatal and perinatal services [65], mother-to-child HIV transmission is frequent in this vulnerable population [27, 69].

One out of four women was ambivalent towards a future pregnancy. Ambivalence regarding pregnancy is common [70]. In our study we observed that women with such intention have more pregnancies than women with a negative intention. The ambivalence with regards to parenthood may lead to inconsistent contraceptive use [71] and may explain the incidence rate that reaches 19 pregnancies per 100 person-years, which was higher than that observed among women with a negative intention. Women who are ambivalent may also have the impression that pregnancy is predetermined or the result of fate [72]. Women with such an attitude towards pregnancy are less likely to use contraception consistently. The use of dual contraception was rare in our study population, particularly in Benin, with less than 20% of the FSWs planning on using hormonal contraception in the next 6 months. Fear and misbeliefs concerning hormonal contraception could explain this low use of contraception [73], but ambivalence towards pregnancy may also play a role.

About 60% of FSWs did not plan to become pregnant in the next 6 months. Despite this clear intention of avoiding pregnancy, the incidence rate of pregnancy (first occurrence, 3-category intention) was 15.2 per 100 person-years in this sub-group. A recent meta-analysis estimated an incidence rate of 27.1 unintended pregnancies per 100 person-years [8]. This result is difficult to compare with ours since it pooled studies from all around the world and they were all, but one, published before 2015. Moreover, the ten studies included in this meta-analysis had low quality data (6 out of ten had a quality assessment of 40% or less) and were using secondary data analyses. Other studies evaluated pregnancy cumulative incidence and measured incidence rate. Some evaluated unintended pregnancies during lifetime [74–77]. This way of measuring unintended pregnancy is not optimal. FSWs have many pregnancies but they have twice more pregnancies before practicing sex work than during sex work practice [43]. When the unintended pregnancy rate is calculated for the entire lifetime, the occurrence of unintended pregnancy during sex work practice is likely to be overestimated. As observed in other studies, a small proportion of women always used a condom with their boyfriend (5%) and less than 1 out of 4 used hormonal contraception. This can explain that 7% of the women who clearly did not plan to get pregnant had an unintended pregnancy during each 6-month period studied. This finding is of a great programmatic interest in that it reflects the core of the unmet contraceptive needs among this sub-population. This is worrisome from the HIV prevention perspective, especially in the context of a high HIV prevalence; one out of four women in our cohort was living with HIV. Moreover, women face more risk of vertical transmission when the pregnancy is unplanned and leads to livebirth [78]. Finally, for some FSWs, even if the pregnancy is unintended, the child is seen as a blessing [79]. It is not all black and white.

We observed that almost two out of five women changed their intention regarding a future pregnancy between the two periods. This instability has previously been documented through qualitative research. Sometimes women want a pregnancy and sometimes not [80]. Fertility desire is not stable [40]. In addition, the length of time on which the fertility desire is measured can also influence the proportion of women who report wanting to be pregnant. For example, a study in Kenya questioned FSWs about their fertility desire. Whereas 26% answered that they wanted children in the future, when asked about their current fertility intent, only 10% were trying to get pregnant [13]. It is important to consider the fluidity of those desires to plan future research and to develop sexual and reproductive services that tailor FSWs’ needs. Fertility desire evaluated over a short period of time seems to give a more accurate picture of the immediate intention.

### Strengths and limitations

One of the limitations of this study is that it was not possible to identify a validated tool that prospectively assesses the intention of becoming pregnant for African FSWs. Based on the existing literature on the subject, we decided to develop our own questionnaire based on a validated tool to estimate that concept and its association with pregnancy. Time limitation made it impossible to test that tool before the study. We used a cluster analysis to overcome that problem.

Pregnancy is a sensitive subject for women practicing sex work. By fear of judgement, FSWs could have underreported pregnancies that occurred between two study visits. To prevent this situation, data collection took place in FSW-friendly clinics, in a confidential setting, and all the women were referred to the study by peer workers. However, this recruitment process could have influenced the profile of the FSWs participating in the study. In fact, FSWs who are more comfortable using the FSW-friendly clinics might have been more inclined to participate. Therefore it might possible to generalize these results only to FSWs who use friendly-clinic services.

Many women were lost to follow-up and that could have induced a selection bias. We therefore compared the women lost to follow-up with those who stayed in the study with regards to pregnancy intention and we observed no difference. We also used IPCW to minimize the selection bias that could have been induced by losses to follow-up in our Cox proportional hazard model. However, such losses to follow-up may have reduced the statistical power of this analysis.

This study also has some strengths. First, the use of a prospective multidimensional measure of the intention to become pregnant gave us a more detailed portrait of FSWs’ intention of having a baby. This is the first time that this level of details is used with regards to FSWs’ pregnancy intention. Moreover, we made sure that we could compare our results to other studies by using simultaneously a known measure with two categories. Also, the categories used for the two measures of intention (three categories and two categories) were exhaustive and mutually exclusive. Secondly, to ensure comprehensive ascertainment of all the pregnancies, we used two types of measure, a pregnancy test, and a questionnaire on pregnancy occurrence in the previous 6 months. The questionnaire was used to make sure that women who terminated their pregnancy or had a miscarriage were also included in the analysis and that we did not underestimate the occurrence of pregnancy. Thirdly, we asked women at two different times about their intention to become pregnant and followed them up. The repeated assessment of the intention and the occurrence of pregnancies gave us the opportunity to observe if their intention materialize and if the intention aligned with the occurrence of a pregnancy. The longitudinal design of our study and the adjustment of the hazard ratio model, despite the reduced statistical power, gave us the opportunity to have a glance at a possible causal effect of the intention on pregnancy occurrence. Finally, we were able to recruit women from two quite different countries in West Africa. This opportunity gave us the possibility to extend our findings to a larger population of FSWs.

## Conclusion

Among FSWs in Benin and Mali, pregnancy intention seems to vary over short periods of time and is associated with pregnancy incidence. FSWs also have similar fertility desires as women who are not FSWs. One of the sustainable development goals is to ensure healthy lives and promote well-being for all [81]. For women, this translates into reducing global maternal mortality and ensuring universal access to sexual and reproductive health-care services by 2030 [82]. In the context of sex work, giving women access to sexual and reproductive health care services is essential to prevent negative impact on the health of the women and their future children. In the context of FSW programs and the adverse health risks associated with sex work, health workers should perform an assessment of pregnancy intention of FSWs at least twice a year. This assessment could also be used to inform and adequately support FSWs through their desire of becoming mothers or help them adequately prevent unintended and/or unwanted pregnancies. Finally, by studying this important topic and discussing those results, we hope to contribute to reduce stigmas surrounding pregnancy in the sex workers population.

## Supplementary Information

**Additional file 1. **Questions used to define each aspect of the intention

**Additional file 2. **Total variation explained by the Cluster analyses and 1-R^2^ ratio.

## Data Availability

The datasets used and analyzed during the current study are available from the corresponding author on reasonable request.

## Abbreviations

aHR: Adjusted hazard ratio
CI: Confidence interval
FCFA: Franc des communautés financières africaines
FSW: Female sex workers
HIV: Human immunodeficiency viruses
HPV: Human papillomavirus
HR: Hazard ratio
IPCW: Inverse probability of censoring weights
NGO: Non-Governmental Organization
