# Supplementary material for: Longitudinal study of pregnancy intention and its association with pregnancy occurrence among female sex workers in Benin and Mali
Source: Reprod Health. 2023 Jan 30;20:25. doi: 10.1186/s12978-023-01565-4 (PMC9887776; doi:10.1186/s12978-023-01565-4)
Supplement: Supplementary file 1 — Additional file 1. Questions used to define each aspect of the intention [file 12978_2023_1565_MOESM1_ESM.docx]

**Additional file**

**Additional file 1.** Questions used to define each aspect of the intention.

| Aspects | Components | Questions | Possible answers |
| --- | --- | --- | --- |
| ***1- Context*** |  |  |  |
|  | Personal circumstance | What is your ideal family size? How many biological children do you have? | Ideal family size minus number of biological children |
|  | Perceived timing | Which statement best describe your thoughts about the possibility of being pregnant in the next six months? | Good timing, ok but not a good timing, bad timing |
| **2- *Stance*** |  |  |  |
|  | Pregnancy desire | Do you want to be pregnant in the next six months? | Yes or no |
|  | Expressed intention | How important it is for you to avoid a pregnancy in the next six months? | Extremely important, really important, important, does not matter, not really important, not important |
| **3- *Behavior*** |  |  |  |
|  | Pre-conceptual preparation 1 | Whom do you plan to get pregnant from? | Client, husband, boyfriend or other |
|  | Pre-conceptual preparation 2 | Did you discuss that project with that person? | No discussion, discussion and disagreement and discussion and agreement |
