# Supplementary material for: Longitudinal study of pregnancy intention and its association with pregnancy occurrence among female sex workers in Benin and Mali
Source: Reprod Health. 2023 Jan 30;20:25. doi: 10.1186/s12978-023-01565-4 (PMC9887776; doi:10.1186/s12978-023-01565-4)
Supplement: Supplementary file 2 — Additional file 2. Total variation explained by the Cluster analyses and 1-R2 ratio. [file 12978_2023_1565_MOESM2_ESM.docx]

**Additional file**

**Additional file 2**. Total variation explained by the Cluster analyses and 1-R^2^ ratio

| Cluster analysis (n= 652) | Number of clusters | Variables | Clusters | R^2^ * | 1-R^2^ ratio ** | Total variation explained *** |
| --- | --- | --- | --- | --- | --- | --- |
| **Option 1** |  |  |  |  |  |  |
|  | 2 | Stance – pregnancy desire |  | 0.0391 | 0.4334 |  |
| 6 variables without restriction |  | Behavior – pre conceptual 1 |  | 0.2119 | 0.2848 |  |
|  |  | Behavior – pre conceptual 2 | 1 | 0.1786 | 0.1610 |  |
|  |  | **Context – personal circumstance** |  | 0.0007 | **0.9905** |  |
|  |  | Context – perceived timing |  | 0.0031 | 0.1393 |  |
|  |  | Stance – express intention | 2 | 0.2077 | 0.1827 | 66.84% |
| **Option 2** |  |  |  |  |  |  |
| 6 variables without restriction | 3 | **Stance – pregnancy desire** |  | 0.0553 | **0.4348** |  |
|  |  | Behavior – pre conceptual 1 |  | 0.3261 | 0.2789 |  |
|  |  | Behavior – pre conceptual 2 | 1 | 0.2295 | 0.1591 |  |
|  |  | Context – perceived timing |  | 0.0179 | 0.1392 |  |
|  |  | Stance – express intention | 2 | 0.2547 | 0.1835 |  |
|  |  | Context – personal circumstance | 3 | 0.0030 | 0.0000 | 83.42% |
| **Option 3** |  |  |  |  |  |  |
|  | 2 | Context – perceived timing |  | 0.0006 | 0.5138 |  |
| 5 variables without restriction |  | Stance – express intention |  | 0.0008 | 0.1866 |  |
| Without Stance – pregnancy desire |  | Behavior – pre conceptual 1 |  | 0.0006 | 0.2173 |  |
|  |  | Behavior – pre conceptual 2 | 1 | 0.0030 | 0.3067 |  |
|  |  | Context – personal circumstance | 2 | 0.0004 | 0.0000 | 75.54% |
| **Option 4** |  |  |  |  |  |  |
| 5 variables with restriction | 3 | Behavior – pre conceptual 1 |  | 0.3260 | 0.1237 |  |
| Without Stance – pregnancy desire |  | Behavior – pre conceptual 2 | 1 | 0.2295 | 0.1082 |  |
|  |  | Context – personal circumstance | 2 | 0.0017 | 0.0000 |  |
|  |  | Context – perceived timing |  | 0.1251 | 0.1563 |  |
|  |  | Stance – express intention | 3 | 0.4426 | 0.2453 | 91.20% |
| **Option 5** |  |  |  |  |  |  |
|  | 2 | Stance – pregnancy desire |  | 0.0553 | 0.4348 |  |
| 5 variables without restriction |  | Behavior – pre conceptual 1 |  | 0.3260 | 0.2789 |  |
| Without Context – personal circumstance |  | Behavior – pre conceptual 2 | 1 | 0.2295 | 0.1591 |  |
|  |  | Context – perceived timing |  | 0.0179 | 0.1392 |  |
|  |  | Stance – express intention | 2 | 0.2547 | 0.1835 | 80.10% |
| **Option 6** |  |  |  |  |  |  |
|  | 3 | Behavior – pre conceptual 1 |  | 0.3460 | 0.1237 |  |
| 5 variables with restriction |  | Behavior – pre conceptual 2 | 1 | 0.3388 | 0.1261 |  |
| Without Context – personal circumstance |  | Context – perceived timing |  | 0.1697 | 0.1647 |  |
|  |  | Stance – express intention | 2 | 0.4426 | 0.2453 |  |
|  |  | Stance – pregnancy desire | 3 | 0.3101 | 0.0000 | 91.20% |
| **Option 7** |  |  |  |  |  |  |
|  | 1 | Behavior – pre conceptual 1 |  | - | - |  |
| 4 variables without restriction |  | Behavior – pre conceptual 2 |  | - | - |  |
| Without Stance – pregnancy desire |  | Context – perceived timing |  | - | - |  |
| Without Context – personal circumstance |  | Stance – express intention | 1 | - | - | 69.43% |
| **Option 8** |  |  |  |  |  |  |
|  | 3 | Behavior – pre conceptual 1 |  | 0.4555 | 0.1531 |  |
| 4 variables with restriction |  | Behavior – pre conceptual 2 | 1 | 0.3587 | 0.1300 |  |
| Without Stance – pregnancy desire |  | Context – perceived timing | 2 | 0.5278 | 0.0000 |  |
| Without Context – personal circumstance |  | Stance – express intention | 3 | 0.5278 | 0.0000 | **95.83%** |

* The R-square value displayed the separation between each variable and the nearest cluster. If the R-square is near 0 then the cluster is well separated

** The 1-R-square ratio represents: (1-R^2^own cluster/1-R^2^nearest cluster).

*** Percentage of the variance in the data accounted for by the clusters.
